# Relevant Feature Integration and Extraction for Single-Trial Motor Imagery Classification

**DOI:** 10.3389/fnins.2017.00371

**Published:** 2017-06-29

**Authors:** Lili Li, Guanghua Xu, Feng Zhang, Jun Xie, Min Li

**Affiliations:** ^1^School of Mechanical Engineering, Xi'an Jiaotong UniversityXi'an, China; ^2^State Key Laboratory for Manufacturing Systems Engineering, Xi'an Jiaotong UniversityXi'an, China

**Keywords:** classification, motor imagery, brain computer interface, single trial, feature extraction

## Abstract

Brain computer interfaces provide a novel channel for the communication between brain and output devices. The effectiveness of the brain computer interface is based on the classification accuracy of single trial brain signals. The common spatial pattern (CSP) algorithm is believed to be an effective algorithm for the classification of single trial brain signals. As the amplitude feature for spatial projection applied by this algorithm is based on a broad frequency bandpass filter (mainly 5–30 Hz) in which the frequency band is often selected by experience, the CSP is sensitive to noise and the influence of other irrelevant information in the selected broad frequency band. In this paper, to improve the CSP, a novel relevant feature integration and extraction algorithm is proposed. Before projecting, we integrated the motor relevant information to suppress the interference of noise and irrelevant information, as well as to improve the spatial difference for projection. The algorithm was evaluated with public datasets. It showed significantly better classification performance with single trial electroencephalography (EEG) data, increasing by 6.8% compared with the CSP.

## Introduction

Brain-computer interface (BCI) is a way of communication that aims to provide a communication path between humans and computers. It directly translates brain activity into a series of control commands. Accordingly, it provides a non-muscular output channel for the brain and communicates with devices directly (Yu et al., 2014). This interface may offer disabled people a great prospect by solely translating their intentions that are reflected in their brain signals into actual instructions (Lemm et al., 2005). In addition, BCI can also be used as a neuro-rehabilitation tool to improve motor and/or cognitive performance of people after neurological diseases, such as stroke (van Dokkum et al., 2015) and tetraplegia (Vuckovic et al., 2015). In the BCI system, several modalities have been used for brain signal acquisition, such as electrocorticographic (ECoG) (Leuthardt et al., 2004), electroencephalography (EEG) (Bennet et al., 2016), magnetoencephalography (MEG) (Sardouie and Shamsollahi, 2012), functional magnetic resonance imaging (fMRI) (Ruiz et al., 2014), functional near-infrared spectroscopy (fNIRS) (Naseer and Hong, 2013, 2015; Hong et al., 2015; Naseer et al., 2016a,b), and intracortical neuronal spikes (Gupta et al., 2016). Among them, because of the real-time, low-cost, portable and noninvasive properties of EEG, it is one of the most convenient means to measure neurophysiological activity in the practical BCI application (Mihajlovic et al., 2015).

Electroencephalography (EEG) modulated by motor imagery (MI) is one of the most studied types of EEG signals of the BCI systems for the similarities of motor-related area involvement with motor execution (Pfurtscheller et al., 1997). MI can be revealed on brain activity patterns of the imagination of a motor action, but without its physical movement. During an MI task, the EEG activity is accompanied by an increase or decrease in the EEG magnitude which is known as an event-related synchronization or desynchronization (ERS/ERD). The ERD and ERS are non-phase-locked modulations of the EEG power, usually confined to a specific frequency band. ERD and ERS have been suggested to reflect the cortical activation and cortical deactivation (Hu et al., 2015). In particular, ERD of μ-rhythm (8–12 Hz) is usually associated with MI (Neuper and Pfurtscheller, 2001; ter Horst et al., 2013). BCI based on MI is an efficient path of rehabilitation, and it achieves excellent findings on complex movement (Qiu et al., 2017).

A big challenge for BCI based on motor imagery is to correctly and efficiently identify and extract subject-specific features from the blurred scalp EEG and translate those features into device commands (Wu et al., 2008). Based on topographic patterns, the Common Spatial Pattern (CSP) has been shown to be very efficient in the establishment of subject-specific discriminative spatial filters (Dornhege et al., 2006). The CSP algorithm decomposes multi-channel EEG from two classes into spatial patterns and enhances the separability between the two classes by diagonalizing the covariance matrix at the same time (Park et al., 2014). However, the conventional CSP algorithm selects multi-channel magnitude features on frequency band, which is selected by experience (Dornhege et al., 2006). As a result, it is sensitive to noise and the influence of other irrelevant information in the selected broad frequency band. Therefore, method for the optimization of the characteristics is urgently needed.

A noteworthy attempt, namely the Common Spatio-Spectral Pattern (CSSP) algorithm has been reported in Lemm et al. (2005). In the CSSP algorithm, the filter is constructed by the method of time-delay embedding. However, the CSSP algorithm limits the flexibility of the filters. The Common Sparse Spectral-Spatial Pattern (CSSSP) performs simultaneous optimization of an arbitrary Finite Impulse Response (FIR) filter (Dornhege et al., 2006). The spectral weighted common spatial pattern (SPEC-CSP) (Tomioka et al., 2006) optimizes the filter in the frequency domain and the spatial filter is an iterative procedure. But, this method is computationally expensive. The Filter Bank Common Spatial Patterns (FBCSP) (Ang et al., 2012) uses mutual information to select the optimal frequency band and time range. Xu applies particle swarm method to optimize frequency band and time interval (Xu et al., 2014). Local temporal correlation common spatial patterns employs local temporal information to estimate covariance matrices instead of Euclidean distance method of CSP (Zhang et al., 2013). The Regularizing Common Spatial Patterns (RCSP) adds a regularization algorithm to the CSP algorithm by a priori knowledge (Lotte and Guan, 2011). However, it does not consider the multivariable nature of the EEG signals, and thus it limits the feasibility of this method.

In this paper, an algorithm designated Spectral Component Common Spatial Pattern (SCCSP) is proposed. It provides a new approach to further improve the classification performance of the motor-imagery-based BCIs. To feature optimize, it focuses on the changes of the amplitude spectrum during motor imagery, and utilizes Independent Components Analysis (ICA) to extract the components from multi-channel amplitude spectrum with the aim of separating motor-relevant and irrelevant information from obscure EEG amplitude features applied by CSP. Accordingly, SCCSP could increase the classification accuracy of single-trial motor imagery EEG by improving the spatial difference of projecting.

## Data acquisition and configuration

**Figure 1:**
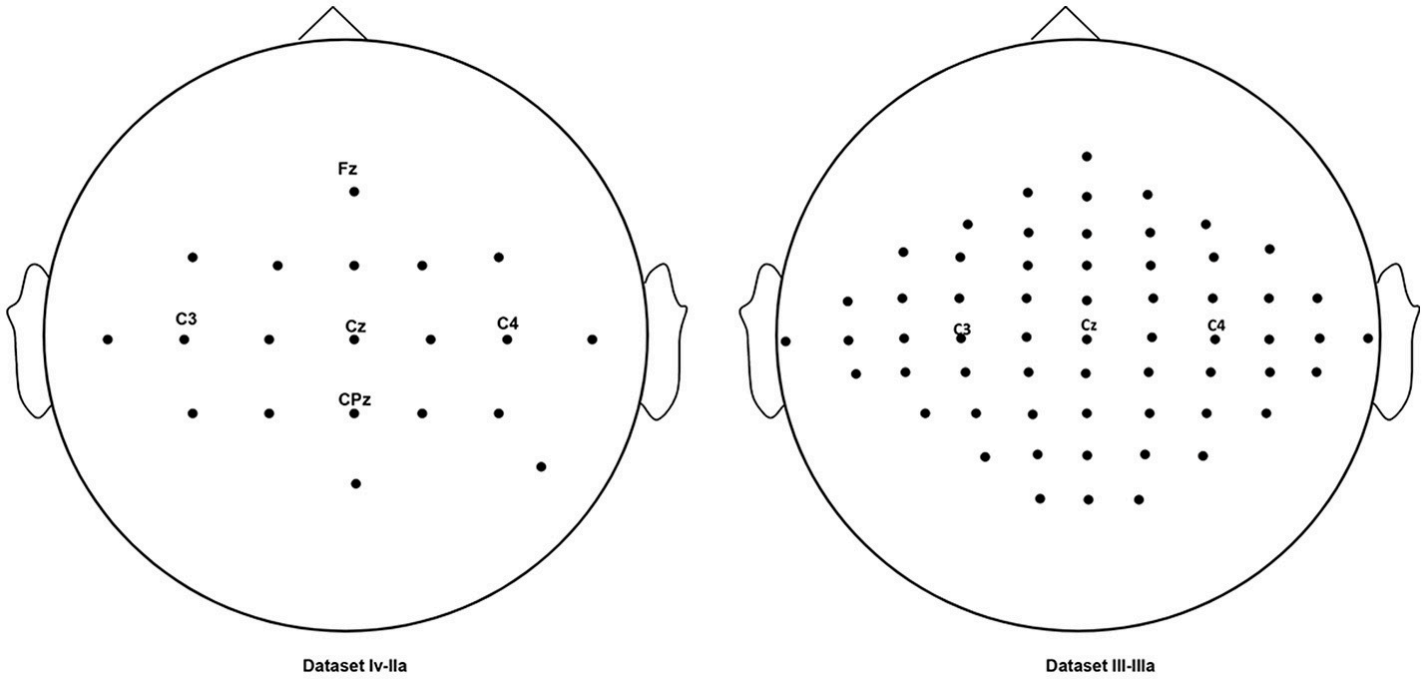
The electrode locations of the datasets.

Two publically available datasets from BCI competitions were collected for the evaluation of the proposed algorithm for motor imagery. For the classification algorithm of CSP is the binary-class classification algorithm, two classes of motor imagery EEG data are collected from the two public datasets. The first public dataset recording the imagination left and right hands movement is collected from the publically available dataset BCI competition IV, dataset IIa (http://bbci.de/competition/iv/), including all 9 subjects. This dataset records EEG with twenty-two electrodes with a sampling rate of 250 Hz. Each trial (experiment) lasts 7.5 s. The subjects imaged movements from *t* = 3 s to *t* = 6 s in trials. Before this period, it is the period for preparation. The second public dataset is the dataset IIIa from the BCI competition III using a 60-channel amplifier with a sampling rate of 250 Hz, including all 3 subjects. The subjects imaged left and right hand movements from *t* = 3 s to *t* = 7 s in trials. Before this period, it is the period for preparation. Both of datasets were online filtered by a bandpass filter and a 50 Hz notchfilter to remove artifacts. A summary of the two datasets is presented in Table 1. The electrodes locations of two datasets are shown in Figure 1.

**Table 1:** Summary of the datasets.

**Dataset**	**Channels**	**MI type**	**Subjects**	**Subject number**	**Trials**
IIa	22	Left vs. right hand	A01	1	138
			A02	2	136
			A03	3	137
			A04	4	129
			A05	5	129
			A06	6	113
			A07	7	133
			A08	8	132
			A09	9	116
IIIa	60	Left vs. right hand	k3b	10	90
			k6b	11	58
			l1b	12	60

## Neurophysiological and mathematical methodology

### Feature extraction and integration

For each part of the human body, there exists a respective region in the primary motor cortex and somatosensory area of the neocortex (Chainay et al., 2004; Blankertz et al., 2008). The imaged part is surrounded by the other regions which represent other parts of the human body. Previous studies (Pei et al., 2005; Byblow et al., 2007) indicated that there was a parallel functional process between the lateral somatosensory area and the mid-central area during activation, indicating the independence of the hand and feet/leg areas during imagery. The inhibition mechanism was independent of the excitation mechanism on the somatosensory area (Ikeda et al., 2000). Accordingly, it is hypothesized that the area which represents the part of the imaged human body is independent of other areas which represent the parts of the un-imaged human body in the neocortex. However, the effect of volume conduction, EEG modulated by MI should be the combination of several independent components. Thus, it is urgent to source separation.

Independent Components Analysis (ICA) is a blind source separation method under the temporal information. It has emerged as a valuable signal processing method for the analysis of multivariate channel data (Woods et al., 2015). Let the time-varying observed signals be *X* = [*x*_1_(*t*), …, *x*_m_(*t*)]^T^, and the *S* = [*s*_1_(*t*), *s*_2_(*t*)…, *s*_n_(*t*)]^T^ *t* = *t*_0_, …, *T*, is matrix that contains unknown pure components, *m* and *n* indicate the channels of the observed signals and components, respectively. ICA assumes that the signal *X* is an instantaneous linear mixture of independent sources:

(1) $$\begin{array}{r} X=ES \end{array}$$

where the matrix *E* of size *m*×*n* is the mixing matrix, whose component represents the linear memoryless mixing channels. To recover all the independent components (ICs) of the observed signals, ICA aims to obtain a de-mixing matrix W with minimal knowledge of *E* and *S*. The recovered signals *U* = (*u*_1_, *u*_2_,…, *u*_n_)^T^ are given by Equation 2 (Monakhova et al., 2015).

(2) $$\begin{array}{r} U=WX \end{array}$$

Therefore, the ICA problem can be restated as the problem of finding *W* such that the sources of *U* are maximally independent.

We focus on the improvement of the classification accuracy based on the oscillatory feature (ERD/ERS). Motor imageries are accompanied by the ERD in specific frequency band (Pfurtscheller and Neuper, 1997), indicating an obvious sinking of the amplitude spectrum. To maximize the separability between classes, the feature extraction and integration algorithm is designed by integrating motor-related information. To suppress the influence of imagination irrelevant information and noise, we want to extract relevant information from blurred feature and integrate imagination related information on multi-channel dimensions into a single dimension. In this paper, we extended the conventional ICA algorithm to the frequency domain, and named it as Spectral Independent Components Analysis (SICA). It is hypothesized that the independent component, which is relevant to the imagination contains most of motor imagination information under the information theory. Information maximization algorithm of ICA (Hansen et al., 2001) was applied, and two independent components, imagination relevant information and imagination irrelevant information, were extracted with the SICA. Accordingly, in SICA, the Equation 2 is reconstructed as below:

(3) $$\begin{array}{l} \left[\begin{array}{l} {\text{u}}_{1}\\ {\text{u}}_{2} \end{array}\right]=\overset{\wedge }{\text{W}}{\text{X}}_{\text{f}}=\overset{\wedge }{\text{W}}[\left|\sum_{t=t_{0}}^{T}{\text{x}}_{\text{1}}e^{-j\omega t}\right|,\left|\sum_{t=t_{0}}^{T}{\text{x}}_{2}e^{-j\omega t}\right|,•••,\\ \;{\left|\sum_{t=t_{0}}^{T}{\text{x}}_{\text{m}}e^{-j\omega t}\right|]}^{\text{T}} \end{array}$$

Where, the matrix of size 2 × *m* is the separation matrix. The matrix *X*_*f*_ of size *m* × *k* is the amplitude spectrum matrix of multi-channel. *k* is the length of amplitude spectrum.

**Figure 2:**
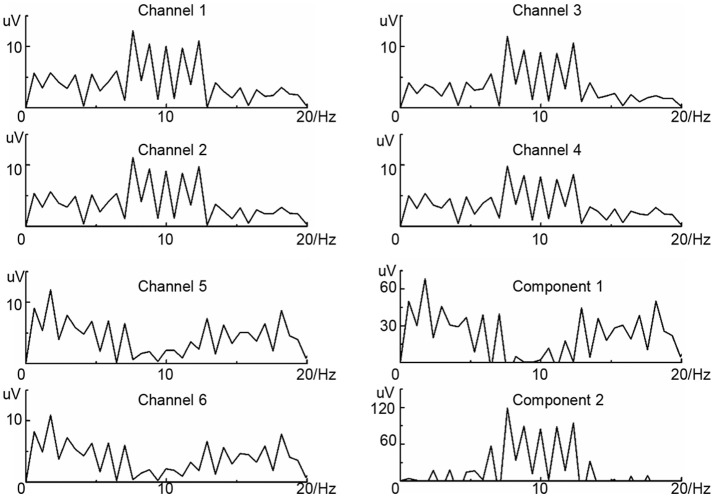
The results of the SICA on simulated data. The frequency spectrum information of six channels are shown from channel 1 to channel 6. Component 1 and component 2 are the ICs extracted.

According to neurophysiological observations, when subjects engage in the unilateral limb imagination, large populations of neurons in the contralateral cortex will be excited, and the scalp EEG rhythm around 10 Hz (μ-rhythm) is significantly suppressed. Namely, the cortex is activated (ERD) (Pfurtscheller and Neuper, 1997). This is a reliable feature of brain activity for BCIs based on motor imagery. For evaluating the effectiveness of SICA algorithm, the 6 channels simulation data of the adult MI EEG without any kinds of mental disease and damage were applied on the same hemisphere. Practically, the ERD often appears on several channels. To imitate this phenomenon, the μ-rhythm on two channels (the 5 and 6th channels) of the simulation data was suppressed. The simulation data of every channel was the sum of the sinusoidal signals with the frequency range from 0 to 20 Hz. The amplitude of the frequencies obtained a greater one on the low frequencies (simulation of real EEG nature), and the sum of maximum and minimum was under 12 uV. The frequency spectrum of simulation data on 6 channels is shown in the Figure 2 after Fast Fourier Transform (FFT). SICA based on information maximization algorithm was used to extract the independent components from the frequency spectrum information of simulation data and the results are illustrated in Figure 2 (component 1 and component 2). The results of the simulation data indicated that the μ-rhythm suppression or activation should be the criterion for the separation of independent components, and the μ-rhythm suppression information was integrated effectively and clearly. Further, SICA is an effective tool in the amplitude spectrum for feature extraction and integration of MI.

### Projecting

The aim of CSP is the maximization of the difference between signals of two classes after feature extraction and integration in this study. *Y*_k_ = [*y*_1_(*t*), *y*_2_(*t*),…, *y*_p_(*t*)]^T^ is defined as the kth time domain feature after feature extraction and integration, where *p* is the number of ICs. The normalization covariance matrices C_1_ and C_2_ of the two classes are calculated using Equation 4. The covariance space C = C_1_ + C_2_ consists of the covariance matrices of the two classes. Whiten the matrix C and receive a matrix P as shown in Equation 5.

(4) $$\begin{array}{r} {{\text{C}}_{1}}_{/2}=\frac{\left({{\text{Y}}_{1}}_{/2}\times {\text{Y}}_{1/2}^{\text{T}}\right)}{\text{trace}({{\text{Y}}_{1}}_{/2}\times {\text{Y}}_{1/2}^{\text{T}})} \end{array}$$

(5) $$\begin{array}{r} PCP^{\text{T}}=\text{I} \end{array}$$

S_1_ and S_2_ are defined as S_1_ = PC_1_P^T^ and S_2_ = PC_2_P^T^, and then calculate the orthogonal matrix R and the diagonal matrix D by singular value decomposition.

(6) $$\begin{array}{r} S_{\text{i}}=RD_{\text{i}}R^{\text{T}} \end{array}$$

Where, i = 1, 2, as I = S_1_ + S_2_, D_2_ = I - D_1_. Therefore, when the eigenvalue of S_i_ (i = 1, 2) is closer to I, the eigenvalue of the other S_i_ (i = 2, 1) is closer to **0**. The difference of the two classes is maximization. The filter is constructed by Equation 7.

(7) $$\begin{array}{r} \text{K}={\text{R}}^{\text{T}}\text{P} \end{array}$$

(8) $$\begin{array}{r} \text{Z}={\text{KY}}_{\text{k}} \end{array}$$

The characteristic for the classifier is calculated by Equation 9.

(9) $$\begin{array}{r} f=\frac{\text{var}({\text{Z}}_{1})}{\text{var}({\text{Z}}_{1})+\text{var}({\text{Z}}_{2})} \end{array}$$

Where, Z_1_ and Z_2_ are the projection of *Y*_k_ by the filters of two classes.

### Data processing

**Figure 3:**
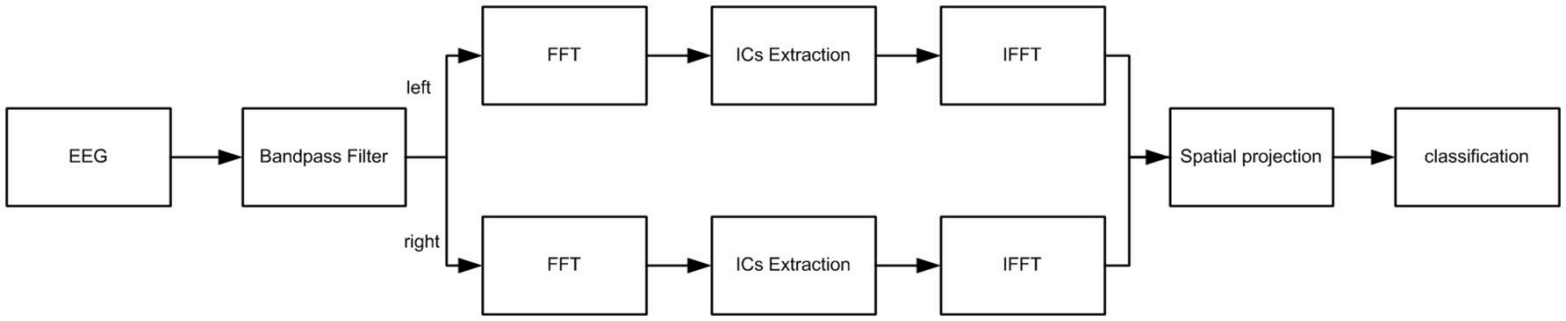
The flow chart for data processing.

All trials were extracted from the two datasets with a bandpass filter of 5–30 Hz by a fourth-order Butterworth filter before analysis. A_k_ = [*a*_1_(*t*), *a*_2_(*t*),…, *a*_g_(*t*)]^T^ *t* = *t*_0_, …, *T* was the kth EEG record, where *g* is the number of electrodes. To suppress the mutual interference of the hemispheres, and to extract and integrate the imagination relevant information by SICA; the EEG data were separated by hemisphere and named as ${\text{A}}_{\text{k}}^{\text{l}}$ and ${\text{A}}_{\text{k}}^{\text{r}}$ in every trial. After fast Fourier transform as illustrated by Equation 10, ${\text{H}}_{\text{k}}^{\text{l}}$ and ${\text{H}}_{\text{k}}^{\text{r}}$ were analyzed by SICA for the feature extraction and integration. Two independent components ${\text{U}}_{1}^{\text{l}\left(\text{r}\right)}$ and ${\text{U}}_{2}^{\text{l}\left(\text{r}\right)}$ which contained imagination relevant or irrelevant information were extracted over each hemisphere. In other words, the imagination relevant information was separated from irrelevant information and integrated together on each hemisphere. After inverse Fourier transform, four temporal components ${\text{Y}}_{1}^{\text{l}}$, ${\text{Y}}_{2}^{\text{l}}$, ${\text{Y}}_{1}^{\text{r}}$, and ${\text{Y}}_{2}^{\text{r}}$ were rearranged as feature matrix according to hemisphere, and the component matrix Y_k_ = [${\text{Y}}_{1}^{\text{l}}$, ${\text{Y}}_{2}^{\text{l}}$, ${\text{Y}}_{1}^{\text{r}}$, ${\text{Y}}_{2}^{\text{r}}$]^T^ was projected. The flow chart of proposed method is illustrated in Figure 3.

(10) $$\begin{array}{r} {\text{H}}_{\text{k}}^{\text{l}(\text{r})}(e^{-j\omega t})=|\overset{T}{\underset{t=t_{0}}{\sum }}{\text{A}}_{\text{k}}^{\text{l}(\text{r})}(t)e^{-j\omega t}| \end{array}$$

Furthermore, whether the proposed method could provide better single trial classification capability than conventional CSP which relied on the bandpass filter was verified by the classification accuracy on twelve subjects of the public datasets. The conventional CSP only applied a bandpass filter from 5 to 30 Hz before projecting. Additionally, the results of the competing feature extraction and integration method, temporal ICA was also reported for comparison. The method named as ICA-CSP which extracted imagination relevant and irrelevant information by conventional temporal ICA before the components were projected. Similarly, ICA-CSP extracted four temporal components and rearranged them according to the hemisphere as SCCSP. The Analytic Common Spatial Patterns (ACSP), CSSSP and the Bilinear Common Spatial Pattern (BCSP) (Yu et al., 2013) and FBCSP were also studied for comparison. The parameters of the FBCSP were the same as the previously reported (Ang et al., 2012). After projecting, a classifier was adopted by LIBSVM (Chang and Lin, 2011) with Radial Basis Function (RBF) by the algorithms. The training and test trials did not overlap on every subject. The numbers of the training and testing trials were half of the whole trials for every subject. The classification performance was evaluated by classification accuracy which is the ratio between the correct number after the classifier and the sum of trials. K-fold cross-validation was applied as cross-validation. The number of K was half of trials in every subject to make sure that every data could be used as the training data and testing data once. K was higher than 10 in all subject. The Lilliefors test was used to evaluate results if they obeyed normal distribution. One-way Analysis of Variance (ANOVA) with repeated measures was applied for statistical analysis of results, and pair *t*-test and least significant difference were used as a *post-hoc* test methods. All calculations were performed in MATLAB.

## Results

**Figure 4:**
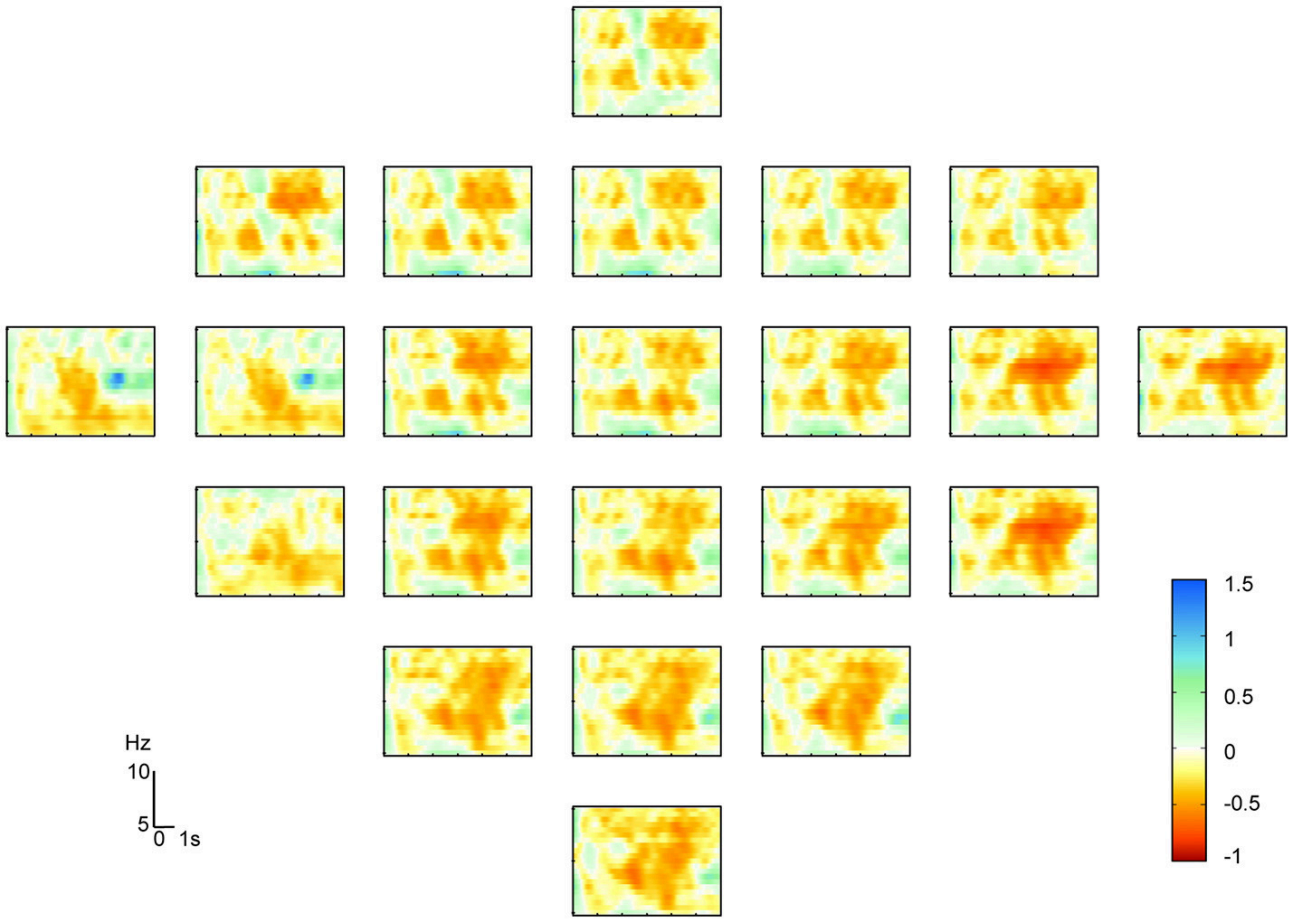
The ERD/ERS maps of subject five on left hand motor imagery. X-axis and Y-axis represent time and frequency, respectively.

Figure 4 shows the ERD/ERS maps at 5–15 Hz of the fifth subject from the dataset IIa during the left hand MI. It indicated that the μ-suppression appeared on several contralateral electrodes. The classification accuracies of the six methods are presented after cross validation in Table 2. They showed that the SCCSP outperformed CSP, ICA-CSP, CSSSP, BCSP and ACSP, achieving 6.8, 3.5, 11.5, 26, and 15.5% higher average classification accuracy than these algorithms, respectively. Among the 12 subjects, SCCSP showed better performance than CSP in 10 subjects. The Lilliefors test showed that the classification accuracy from six algorithms obeyed the normal distribution. The probabilities were 0.1852, 0.5, 0.3136, 0.2141, 0.5, and 0.3909 for the CSP, SCCSP, ICA-CSP, CSSSP, BCSP, and ACSP, respectively. ANOVA indicated that there was significant difference among the six algorithms [*F*_(1, 72)_ = 8.53, *p* < 0.001]. Moreover, the paired *t*-test showed that the better performances of SCCSP over CSP (*p* < 0.05), ICA-CSP (*p* < 0.05), CSSSP (*p* < 0.05), BCSP (*p* < 0.001) and ACSP (*p* < 0.001) were significant. Least significant difference, used as *post-hoc* test, showed that the better performances of SCCSP over CSSSP, BCSP, and ACSP were significant at 0.05 level. Additionally, ICA-CSP achieved 3.3% higher average classification accuracy than CSP. The kappa value was also applied to evaluate the consistency of classification performance.

(11) $$\begin{array}{r} kappa=\frac{p_{\text{o}}-p_{e}}{1-p_{e}} \end{array}$$

**Table 2:** Classification accuracies (%) of subjects.

**Dataset**	**Subjects**	**CSP Mean (std)**	**SCCSP Mean (std)**	**ICA-CSP Mean (std)**	**CSSSP (Yu et al., 2013)**	**BCSP (Yu et al., 2013)**	**ACSP (Yu et al., 2013)**
IIa	A01	84.3 (1.58)	87.1 (2.63)	85.2 (3.05)	86.1	70.8	90.2
	A02	79.4 (3.27)	86.8 (3.62)	81.0 (2.63)	52.0	50	52.0
	A03	82.4 (2.89)	89.7 (1.39)	89.5 (3.56)	86.1	61.8	95.1
	A04	82.3 (3.15)	83.9 (1.94)	81.1 (2.73)	65.9	55.5	69.4
	A05	89.1 (2.34)	90.6 (2.75)	86.8 (1.99)	68.0	49.3	56.9
	A06	83.9 (2.78)	83.9 (3.11)	91.9 (4.53)	66.6	56.2	70.1
	A07	74.2 (2.92)	86.4 (2.84)	80.5 (3.50)	75.0	57.6	78.4
	A08	83.3 (2.59)	89.4 (2.70)	89.0 (3.15)	95.1	63.1	97.2
	A09	74.1 (2.22)	96.3 (8.24)	76.8 (4.99)	93.0	76.3	91.6
IIIa	k3b	93.5 (2.76)	91.3 (2.01)	87.5 (2.50)	95.5	78.8	76.6
	k6b	80.0 (5.96)	90.0 (2.97)	80.0 (4.39)	55.1	63.7	56.8
	l1b	83.3 (2.98)	96.7 (6.05)	100.0 (2.57)	95.0	76.6	51.6
Average		82.5 (3.0)	89.3 (3.4)	85.8 (3.3)	77.8 (16.0)	63.3 (10.3)	73.8 (17.0)

Where, *p*_o_ is the classification accuracy; *p*_e_ denotes the probability of expected agreement. The results of the kappa values are listed in Table 3. The SCCSP outperformed CSP, ICA-CSP and FBCSP, achieving 0.247, 0.094, and 0.109 higher average kappa value than these algorithms, respectively. The Lilliefors test showed that the kappa value from these algorithms followed the normal distribution. The probabilities were 0.5, 0.3573, 0.5, and 0.076 for the CSP, SCCSP, ICA-CSP, and FBCSP, respectively. The ANOVA indicated that there was significant difference among these algorithms [*F*_(1, 36)_ = 5.99, *p* < 0.05] in the kappa value. The paired *t*-test showed that the better performances of the SCCSP over CSP (*p* < 0.001) and ICA-CSP (*p* < 0.05) were significant. The better performance of the ICA-CSP over CSP (*p* < 0.001) was significant. Moreover, the probability of SCCSP performance over FBCSP was 0.08. Least significant difference, used as *post-hoc* test, showed that the better performance of SCCSP over CSP was significant at 0.05 level.

**Table 3:** Kappa scores of BCI competition IV dataset IIa.

**Dataset**	**Subjects**	**CSP Mean (std)**	**SCCSP Mean (std)**	**ICA-CSP Mean (std)**	**FBCSP (Ang et al., 2011)**
IIa	A01	0.556 (0.0316)	0.664 (0.0526)	0.687 (0.0579)	0.747
	A02	0.599 (0.0654)	0.776 (0.0724)	0.689 (0.0359)	0.416
	A03	0.539 (0.0579)	0.776 (0.0277)	0.560 (0.0712)	0.824
	A04	0.419 (0.0629)	0.732 (0.0388)	0.771 (0.0546)	0.400
	A05	0.656 (0.0469)	0.838 (0.0549)	0.742 (0.0399)	0.608
	A06	0.490 (0.0556)	0.701 (0.0622)	0.607 (0.0906)	0.309
	A07	0.430 (0.0585)	0.758 (0.0568)	0.668 (0.0700)	0.849
	A08	0.411 (0.0518)	0.735 (0.0540)	0.615 (0.0631)	0.787
	A09	0.372 (0.0455)	0.717 (0.1648)	0.507 (0.0998)	0.772
Average		0.497 (0.0528)	0.744 (0.0649)	0.650 (0.0648)	0.635 (0.208)

**Figure 5:**
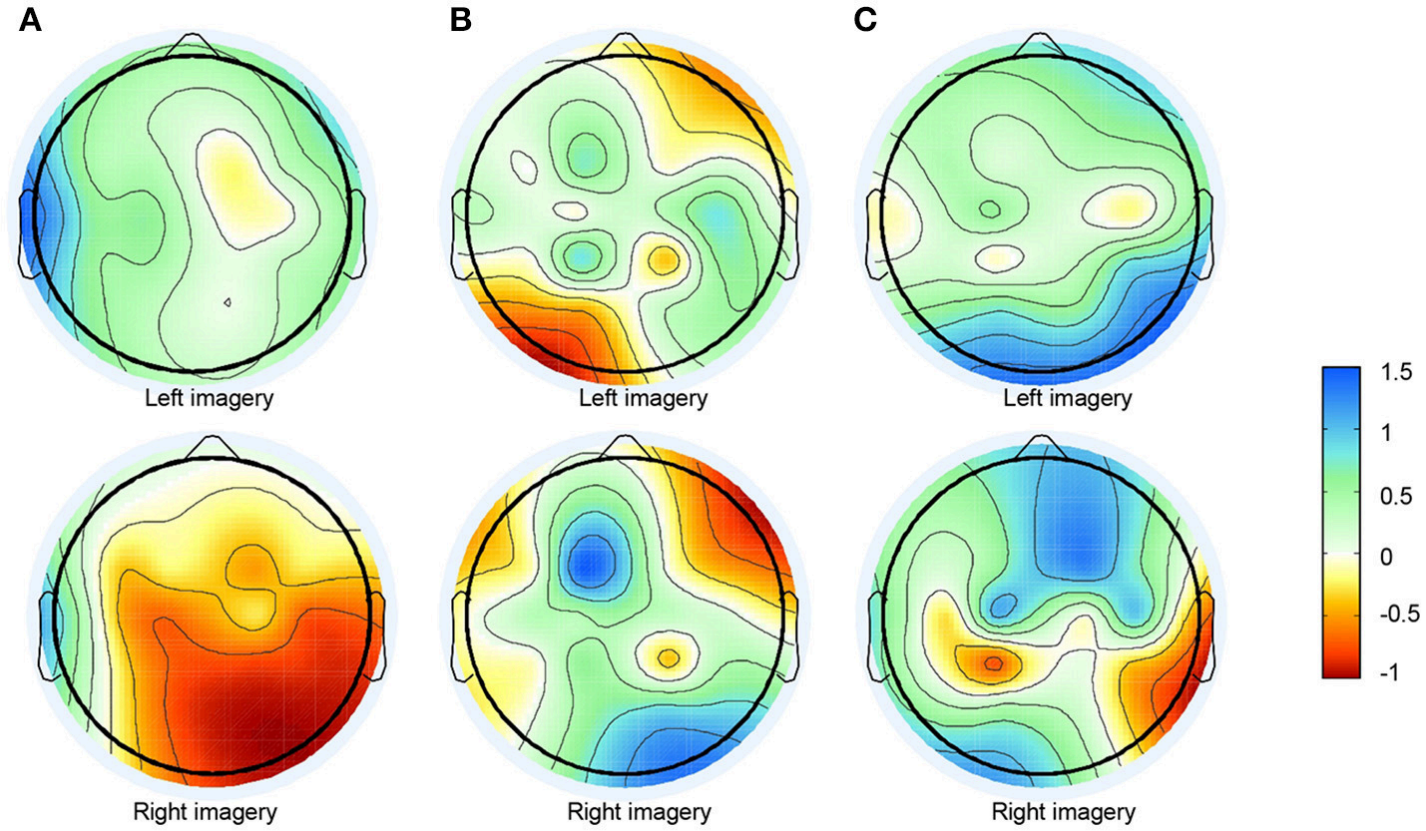
Topographical view of feature extraction algorithm's results. **(A)** Topographical view of average time-frequency representation of ERD/ERS values of hand imagery in 5–15 Hz on the fifth subject. **(B)** Topographic distribution of average power after bandpass filter from 5 to 30 Hz. **(C)** Topographic distribution of average power after feature extraction and integration method.

The feature extraction and integration result of subject 5 is presented in Figure 5. The result presented in Figure 5A shows the topographical view of the average time-frequency representation of ERD/ERS values in μ-rhythm during hand imagery. The result in Figure 5B shows the filtered result by the bandpass filter in CSP. Figure 5C reveals the result obtained by feature extraction and integration algorithm proposed where the components were converted by matrix *W*. The result in Figure 5A was consistent with the fact that the EEG suppressions were contralateral to the imagined hand movement (Pfurtscheller and da Silva, 1999).

**Figure 6:**
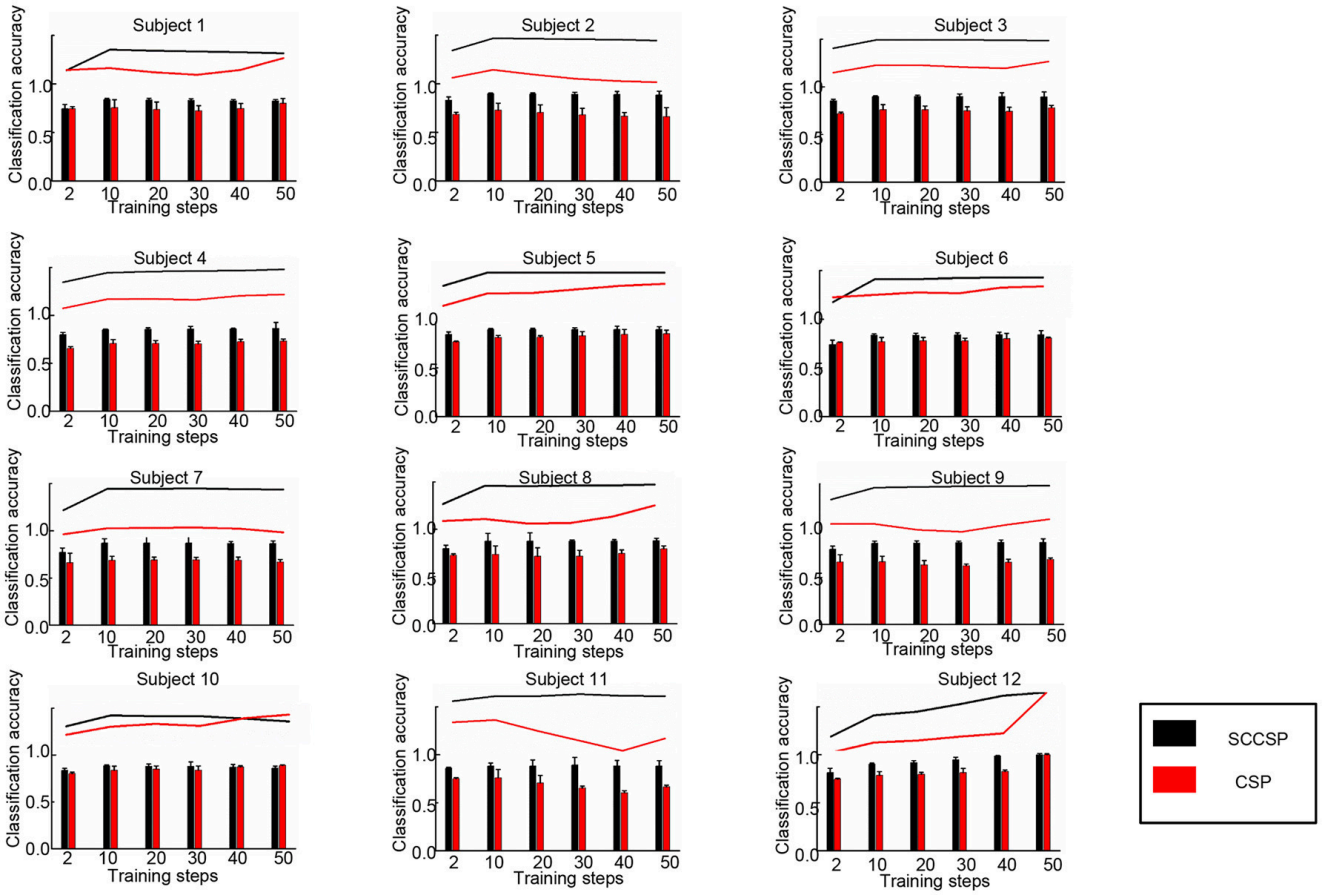
Classification accuracy with varied training datasets from 2 to 50 of the classifier in 12 subjects.

**Figure 7:**
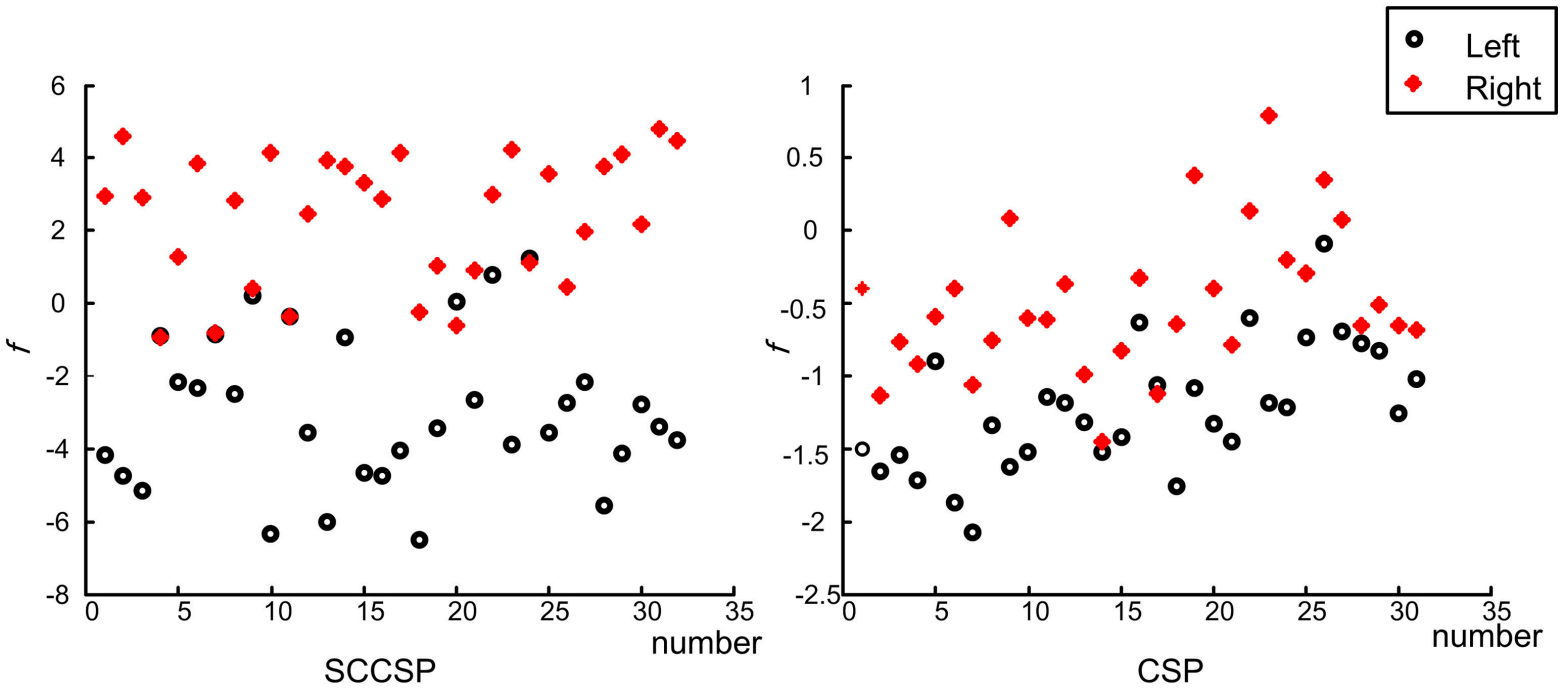
The fifth subject's classification result of two classes on *f*. The circles and crosses indicated the left and right motor imagery.

**Figure 8:**
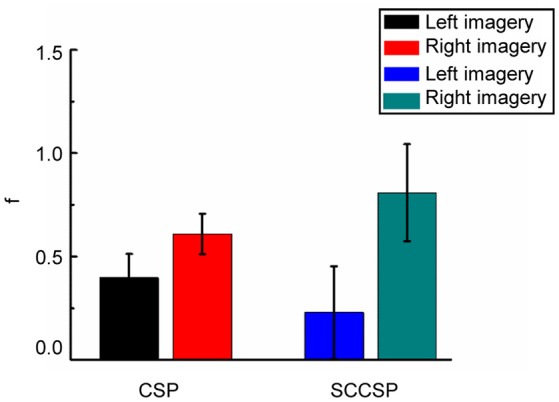
Statistical results of f under SCCSP and CSP on two classes.

To study the stability of the SCCSP, the number of the trials for training the classifier was varied from 2 to 50 with about 10 steps. The results of classification accuracy with error bar for every step are presented in Figure 6 after cross validation. The average classification accuracy and standard deviation of accuracy of the SCCSP and CSP were calculated and are shown in Table 4. The SCCSP achieved 12.1% higher average accuracy than CSP. The Lilliefors test shows that the average classification accuracy from these algorithms obeyed the normal distribution. The probabilities were 0.5 and 0.5 for CSP and SCCSP, respectively. The ANOVA results indicated that there was significant difference between these algorithms [*F*_(1, 24)_ = 35.97, *p* < 0.001] for classification accuracy. The paired *t*-test showed that the better performance of SCCSP over CSP was significant (*p* < 0.001). Furthermore, SCCSP had a smaller average standard deviation of classification accuracy than CSP. A classification of the *f* in Equation 9 of subject 5 is shown in Figure 7 for visualization. The statistical results of the *f* under the SCCSP and CSP are shown in Figure 8. A paired *t*-test analysis showed that the SCCSP achieved a higher difference between the two classes than CSP (*p* < 0.05). For quantitative analysis, the within-class distance *B* and between-class distance *D* were applied.

(12) $$\begin{array}{r} B=\frac{1}{\text{M}}\underset{k\in {\text{C}}_{1}/\text{C}2}{\sum }||d_{k}|| \end{array}$$

(13) $$\begin{array}{r} D\left({\text{C}}_{1}{\text{C}}_{2}\right)=\frac{1}{{\text{N}}_{1}{\text{N}}_{2}}\underset{i\in {\text{C}}_{1}}{\sum }\underset{j\in {\text{C}}_{2}}{\sum }||{d_{i}}_{j}|| \end{array}$$

(14) $$\begin{array}{r} \lambda =\frac{B_{1}+B_{2}}{D} \end{array}$$

**Table 4:** Average classification accuracies (%) and standard deviation of accuracy (%) of the datasets IIa and IIIa in different steps.

**Dataset**	**Subjects**	**CSP Mean (std)**	**SCCSP Mean (std)**
IIa	A01	75.2 (2.7)	81.7 (3.6)
	A02	69.0 (2.5)	88.3 (2.5)
	A03	75.3 (2.0)	89.1 (1.8)
	A04	70.48 (2.7)	84.8 (2.5)
	A05	82.1 (3.0)	88.8 (2.1)
	A06	78.3 (1.7)	82.3 (4.1)
	A07	68.3 (1.3)	85.2 (3.9)
	A08	73.8 (2.9)	86.4 (3.3)
	A09	65.9 (2.4)	86.0 (2.9)
IIIa	k3b	82.8 (8.9)	92.9 (6.8)
	k6b	69.0 (6.0)	88.2 (1.2)
	l1b	84.8 (3.0)	86.9 (1.8)
Average		74.6 (3.3)	86.7 (3.0)

Where, || *d*_k_ || denotes the Euclidean distance between the *f*_k_ and the gravity in C_1_ or C_2_. || *d*_ij_ || is the Euclidean distance between the *f*_i_ in C_1_ and the *f*_j_ in C_2_. To evaluate the difference between the two classes, the ratio of within-class distance and between-class distance λ is derived using Equation 14. A lower λ indicated a greater separability between classes. As a result, the SCCSP achieved nearly twenty times reduction of λ compared to the conventional CSP algorithm, on average.

## Discussion and conclusion

Before the onset of motor imagery and execution, somatosensory area which is a part of the posterior parietal lobe needed some information, such as location, which comes from proprioception and visual area, etc. The prefrontal lobe and posterior parietal lobe determine and control movements. The axons of the prefrontal lobe and posterior parietal lobe concentrate on the Brodmann area 6 of which including the Supplementary Motor Area (SMA) and the Premotor Area (PMA). Most of the corticospinal tracts connect with the efferent fibers of the Brodmann area 6 which encodes the movement and primary motor cortex. The independence discussion of the inhibition mechanism and excitation mechanism on different motor-function area of somatosensory area (Ikeda et al., 2000; Pei et al., 2005) provided a great possibility of activation independence on function areas which represent different parts of body in the primary motor cortex. This reveals that EEG of one-task mental motor imagery should be the combination of time and spatial independent sources on motor-related areas.

In this paper, we extended the temporal ICA to amplitude spectrum analysis. A novel SCCSP algorithm for motor imagery classification based on SICA was proposed. This SCCSP method provided greater classification accuracy than CSP, ICA-CSP, CSSSP, BCSP, and ACSP. The kappa results also exhibited a better performance than CSP, ICS-CSP, and FBCSP. SICA is the extension of blind source separation. Therefore, the better classification performances of SCCSP and ICA-CSP may indicate that the time-frequency independence nature of motor-related sources in this experiment. Moreover, the greater average classification accuracy of SCCSP than ICA-CSP may show a possibility of greater separability or independence on frequency domain. In practice, the channels which reveal μ-suppression varied with trials. For the volume conduction, the suppression appeared in a wide region. This was a challenge to improve the spatial separability of the features. However, the algorithms for projecting were sensitive to the arrangement of feature, spatial distribution. Under SCCSP, a feature extraction and integration method based on SICA was applied. This method can extract the relevant imagination information into one component. That is, the integration of the feature algorithm could separate the motor relevant information from blurred data on multi-channel, concentrate relevant feature, suppress the influence of other region which represent other un-imaged parts of the body, and noise, and enlarge the spatial distribution separability of the features. The pure bandpass filter applied by CSP only suppressed the interference of other frequencies, while the information of other irrelevant function areas and noise remained in the frequency band selected. The results presented in Figures 5B,C illustrate that the proposed algorithm obtained a greater spatial separability, while the information extracted by bandpass filter was obscure. The greater spatial separability extracted by the feature extraction and integration algorithm was favorable for improving the classification accuracy. Therefore, this SCCSP can reduce the interferences both in the other frequency bands and in the frequency band selected to improve classification accuracies. Moreover, the results of SCCSP and spatio-spectral filter selection method by cognitive fuzzy inference system (SCIF) (Das et al., 2016) indicated that there was 3.3% accuracy increase of SCCSP over SCIF on dataset IIa. Though there were different datasets, it was comparability. SCCSP achieved 5.18% higher average accuracy than the dynamic frequency feature selection method mentioned in Luo et al. (2016). Therefore, SCCSP achieve a better performance on MI classification.

SCCSP provided a lower average standard deviation of classification accuracy than almost all other methods. The statistical results of classification accuracy and kappa values indicated that the feature extraction and integration of SCCSP should be individual variability and adaptability. That is, SCCSP can decrease the individual difference. In Table 3, ICA-CSP and SCCSP both achieved significantly higher performances regarding the kappa value. Therefore, ICA is an efficient feature extraction algorithm to improve the spatial separability of features. The results presented in Figure 6 and Table 4 illustrated that SCCSP achieves greater average classification accuracy and a smaller standard deviation compared with CSP, simultaneously. The curve of the classification accuracy on SCCSP was steadier than CSP, and it obtained greater classification accuracy under the small training dataset. Therefore, SCCSP was less affected by the number of training datasets. This is very important for BCI application. Figure 7, **8** illustrated that the SCCSP algorithm obtained a greater separability between classes after classifier. The statistical results of λ indicated that SCCSP can improve the classification accuracy by improving the separability of classes. In BCI applications, there existed multi-class classification problem. The algorithms by spatial projection applied multiple binary-class classification to achieve multi-class classification, such as CSP. Thus, SCCSP can obey this way to classify multi-classes. In this way, one class can be seperated from other classes. Moreover, SCCSP applied SICA and spatial projection to obtain the spatial filter, and furter, the method may also be extended to other higher time resolution signal modalities analysis, such as fNIRS.

In conclusion, in this study, SCCSP has been introduced to the CSP family. This algorithm naturally integrates the relevant information and suppresses the influence of irrelevant information. The accuracy merits of SCCSP as supplemental to the broadband CSP filtering have been attentively validated on the public datasets of motor imagery EEG signals. The quantitative comparisons suggest superior discrimination and stable capability of the proposed method over the conventional CSP. Moreover, the test with varied training datasets shows excellent performance in small training datasets, and this is important in practical application. However, SCCSP spends longer time than CSP. This algorithm needed to be optimized. The SCCSP is affected by the μ-rhythm oscillation on the homolateral hemisphere. For improving the classification accuracy, the suppressed method of the homolateral hemisphere influence should be studied in further studies. In the future, we plan to study the SCCSP for multi class classification.

## Author contributions

Analyzed the data: LL and FZ. Wrote the paper: LL. Contributed materials and analysis tools: LL and GX. Language correction: JX and ML.

### Conflict of interest statement

The authors declare that the research was conducted in the absence of any commercial or financial relationships that could be construed as a potential conflict of interest.
